# Real-world treatment patterns and clinical outcomes among elderly patients with locoregionally advanced head and neck squamous cell carcinoma in the United States

**DOI:** 10.3389/fonc.2025.1606990

**Published:** 2025-09-10

**Authors:** Dandan Zheng, Su Zhang, Behzad Bidadi, Nati Lerman, Yan Song, Rui Song, Jiayang Li, Anyu Zhu, Yuexin Tang, James Signorovitch, Sanjay Merchant, Glenn J. Hanna

**Affiliations:** 1 Outcomes Research, Merck & Co., Inc., Rahway, NJ, United States; 2 Analysis Group, Inc., Boston, MA, United States; 3 Oncology Late Stage Development, Merck & Co., Inc., Rahway, NJ, United States; 4 Center for Head and Neck Oncology, Dana-Farber Cancer Institute, Boston, MA, United States

**Keywords:** event-free survival, head and neck squamous cell carcinoma, locoregionally advanced, medicare, overall survival, real world, treatment patterns

## Abstract

**Introduction:**

Multimodal therapy, including resection followed by adjuvant radiotherapy (RT) ± systemic therapy (ST) or definitive RT ± ST, is typically recommended for patients with locoregionally advanced head and neck squamous cell carcinoma (LA HNSCC) treated with curative intent. We assessed the real-world use of various treatment modalities and associated survival outcomes among elderly patients with LA HNSCC who received surgical or non-surgical primary treatment.

**Methods:**

Linked SEER-Medicare data were used in this retrospective cohort study. Patients with newly diagnosed stage III-IVB LA HNSCC (larynx, hypopharynx, oral cavity, or oropharynx) from 2007–2019 who received primary treatment within 4 months after initial diagnosis were included. Real-world event-free survival (rwEFS) and overall survival (rwOS) from the index date (primary treatment initiation date) were described using Kaplan-Meier estimates. The correlation between rwEFS and rwOS was investigated by normal scores rank. Landmark analysis was conducted using Cox proportional hazards models to compare rwOS between patients with versus without recurrence in the first year after primary treatment initiation.

**Results:**

Of 2180 patients meeting the selection criteria, 626 and 1554 were categorized into the resected and unresected cohorts, respectively (median follow-up: 20.8 and 22.6 months). Overall, the mean age at initial diagnosis was 74.3 years, 65.9% were male, and 81.7% were White. More than half (56.3%) of the patients with resected tumors received RT ± ST post-surgery while nearly two-thirds (64.9%) of those with unresected tumors received definitive RT+ST. The resected cohort had a median rwEFS of 7.8 (95% confidence interval [CI]: 6.4, 8.7) months and a median rwOS of 31.4 (95% CI: 25.2, 40.1) months. The unresected cohort had a median rwEFS of 10.0 (95% CI: 9.4, 10.9) months and a median rwOS of 32.4 (95% CI: 28.5, 36.7) months. There was a significant positive correlation between rwEFS and rwOS for both the resected (*r* [95% CI]: 0.69 [0.63, 0.73]) and unresected (0.68 [0.63, 0.73]) cohorts (both *p*<0.001). In the resected cohort, there was a trend of lower rwOS among patients who experienced recurrence within the first year post-index as compared with those without recurrence (adjusted hazard ratio [95% CI]: 1.31 [0.96, 1.80]), whereas in the unresected cohort, the association was significant (1.91 [1.60, 2.29]).

**Conclusion:**

In elderly patients with LA HNSCC, surgery followed by RT and definitive RT+ST were the most common treatment modalities in the resected and unresected cohorts, respectively. The suboptimal real-world survival of both groups highlights the significant unmet need for more effective therapies. The positive associations between rwEFS and rwOS in both the resected and unresected cohorts support EFS as a predictor of OS when OS data are immature in LA HNSCC.

## 1 Introduction

Head and neck cancer is a heterogeneous group of tumors affecting the oral cavity, oropharynx, hypopharynx, larynx, and several other anatomical subsites (1), and comprise approximately 4% of all cancers in the United States (US) and 3% of all cancer-related deaths worldwide (2). Squamous cell carcinoma accounts for over 90% of head and neck cancer cases (3, 4). Approximately 60% of patients with head and neck squamous cell carcinoma (HNSCC) present with locoregionally advanced (LA) disease at diagnosis (3), which is associated with higher risk of local recurrence and a poorer prognosis (5–7). Approximately 30% of people with HNSCC are aged 70 years or older, and the incidence of HNSCC among elderly adults is expected to increase as lifespans continue to lengthen (8, 9).

Treatment of HNSCC is guided by patient and disease characteristics, including the primary tumor location, stage at diagnosis, and the patient’s age, comorbidities, and preferences (3). For patients presenting with LA HNSCC, the standard treatment approach includes surgical resection followed by radiotherapy (RT) or concurrent chemoradiotherapy (CRT), or definitive CRT when resection is less ideal (3, 10). However, the 5-year survival rates of patients with LA HNSCC who receive these treatments are suboptimal, below 60% in the overall population (5–7, 11), highlighting the unmet need for more effective treatment strategies. Additionally, elderly patients with LA HNSCC are at higher risk of morbidity (i.e., complications, toxicity) or mortality from standard treatment approaches (9).

Overall survival (OS) is one of the most reliable and valuable outcomes in cancer clinical trials (12, 13). However, assessing OS requires a large sample with an extended follow-up period to observe the death events and detect statistical differences, delaying treatment availability for patients. Alternatively, time-to-event outcomes such as event-free survival (EFS), which includes disease recurrence events that occur more frequently and earlier than death, enable trial designs with smaller sample sizes and shorter follow-up duration (14, 15). In particular, EFS has been widely used as an outcome across trials of early-stage tumors when OS is not mature, including in the KEYNOTE-522 trial in early-stage breast cancer and the MATTERHORN trial in resectable gastric cancer (16, 17). Therefore, assessing correlation between EFS and OS in LA HNSCC can help to infer the long-term clinical benefit of treatment, enhancing the clinical relevance of EFS in LA HNSCC and supplementing existing studies assessing the surrogacy of EFS for OS in HNSCC (11, 18). Further, identifying risk of recurrence in LA HNSCC can be meaningful due to the high morbidity and reduced quality of life when locoregional disease recurs.

To support the development of and access to novel treatments for LA HNSCC, it is important to define the unmet medical needs by assessing the treatments used and associated clinical outcomes in the real world. This study used real world data to examine the primary treatment patterns (surgical vs. non-surgical) among elderly adults with LA HNSCC, and to evaluate the real-world OS (rwOS), real-world EFS (rwEFS), and cumulative risk of recurrence for patients with resected and unresected LA HNSCC. Additionally, this study explored baseline factors associated with rwEFS and rwOS and assessed whether rwEFS is a reliable predictor of rwOS in these patient populations.

## 2 Methods

### 2.1 Data source and patient selection

This retrospective, observational study used the Surveillance, Epidemiology, and End Results (SEER)-Medicare database, with SEER cases between 2007–2019 and linked Medicare claims between 2006-2020, to identify patients with newly diagnosed LA HNSCC who received primary treatments for HNSCC within 4 months of initial diagnosis. The SEER-Medicare database links two large US population-based sources of data that provide detailed information about Medicare beneficiaries with cancer, including healthcare services, diagnoses, treatments, demographics, and cause of death (19). The data are limited and in compliance with the Health Insurance Portability and Accountability Act (HIPAA) and its implementing regulations. Thus, this study received an exemption from full review from the Advarra Institutional Review Board on March 11, 2022 (Reference number: Pro00061935).

**Figure 1:**
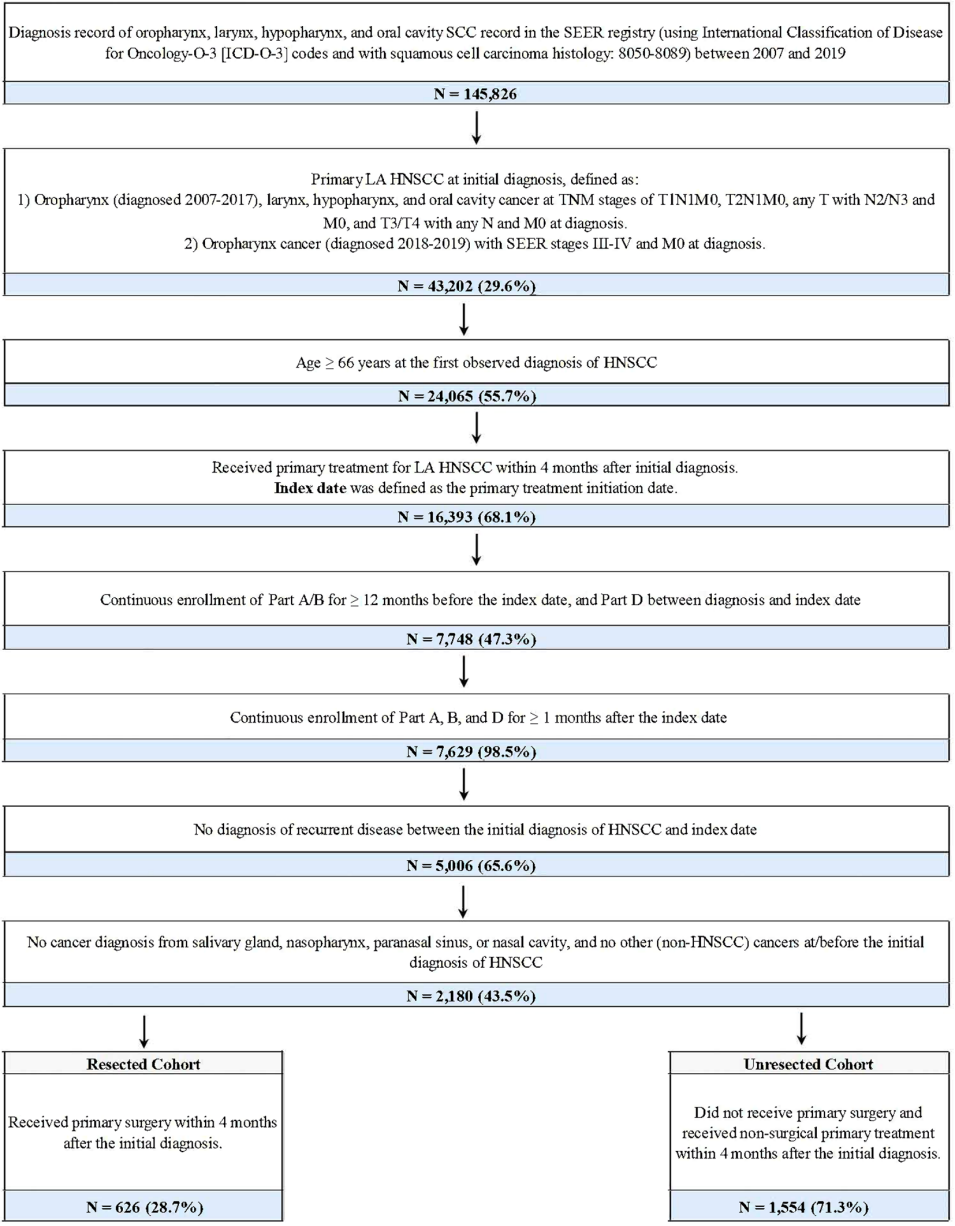
Sample selection and creation of patient cohorts^a^. AJCC, American Joint Committee on Cancer; HPV, human papillomavirus; LA HNSCC, locoregionally advanced head and neck squamous cell carcinoma; SCC, squamous cell carcinoma; SEER, Surveillance, Epidemiology, and End Results; TNM, Tumor/Node/Metastasis. ^a^The AJCC 6/7/8th edition TNM staging system and the stage group information from SEER were applied for patients diagnosed from 2007-2019. For patients diagnosed with oropharynx cancer in 2018 or 2019, SEER stage groups based on AJCC 8th edition TNM were used to identify LA disease regardless of HPV status as HPV status was not available in the data. In the SEER registry, for a given diagnosis, pathological information was used to derive the TNM stage if it was available; otherwise, clinical information was used instead.

Patients were included if they were newly diagnosed with primary LA HNSCC of the oropharynx, larynx, hypopharynx, or oral cavity between 2007 and 2019, were 66 years or older at diagnosis, and underwent primary treatment for LA HNSCC within four months after initial diagnosis. Further details of patient selection are shown in **Figure 1**.

### 2.2 Study cohorts

Patients who met all eligibility criteria were included and assigned to the resected cohort or the unresected cohort based on their primary treatment modality. The resected cohort was defined as patients who received surgery within 4 months after the initial diagnosis. The unresected cohort was defined as patients who did not receive surgery and were treated with radiation therapy and/or systemic therapy within 4 months after the initial diagnosis.

### 2.3 Study outcomes

Baseline patient demographic and clinical characteristics were collected in the 12 months on or prior to the date of primary treatment initiation (index date) for the resected and unresected cohorts. Primary treatment patterns after initial diagnosis were also identified for each cohort.

Clinical outcomes assessed from the index date included rwEFS, rwOS, and time to recurrence. rwEFS was defined as time from the index date to first recurrence or death, whichever occurred first. Recurrence was indicated by ≥2 visits with a diagnosis code for secondary malignancy that were 30 days or less apart and at least 30 days after the initiation of primary treatment, and/or any additional treatment initiation for HNSCC. A sensitivity analysis defining rwEFS as the time from the index date to the earliest of the initiation of the next line of therapy or death was also performed.

For the description of rwOS and the correlation analysis between rwOS and rwEFS, rwOS was defined as time from primary treatment initiation to death due to any cause. For the landmark analysis (described in the Statistical Analysis section), rwOS was defined as time from the corresponding landmark point to death. Patients were censored either at loss to follow-up; end of Medicare Part A, B, or D eligibility; or the end of the study period (12/31/2020), whichever occurred first.

### 2.4 Statistical analysis

Baseline characteristics were separately described for the resected and unresected cohorts. Means and standard deviations (SD) and/or medians and interquartile ranges (IQR) were reported for continuous variables; frequencies and percentages were reported for categorical variables. Primary treatment patterns and the proportions of patients on each primary treatment were summarized within each cohort.

rwEFS and rwOS from the index date were separately described for the resected and unresected cohorts using Kaplan-Meier (KM) curves. Cumulative incidence of recurrence accounting for competing risk with death was estimated. Stratified analyses describing the rwEFS, rwOS, and cumulative incidence of recurrence by tumor sites within each cohort were also performed. Cox proportional hazard models were utilized to assess the association between baseline factors and rwEFS and rwOS, including clinical prognostic factors such as age at index, sex, race, region at diagnosis, stage at diagnosis, primary cancer site at diagnosis, baseline Charlson Comorbidity Index (CCI) score, and primary treatment received. Hazard ratios (HRs) and the corresponding 95% confidence intervals (CIs) were estimated.

The normal scores rank correlation between rwEFS and rwOS from the date of primary treatment initiation was separately estimated for the resected and unresected cohorts. KM curves were plotted to compare the subsequent OS of patients who were event-free (i.e., without recurrence events) in the first year after primary treatment initiation with those who were not (i.e., with recurrence). *P*-values from the log-rank test were reported; a *p*<0.05 was considered statistically significant. Cox proportional hazard models were conducted to assess the association between recurrence within the first year after primary treatment initiation and subsequent OS, adjusting for age at index, sex, race, geographic region at diagnosis, stage at diagnosis, primary cancer site at diagnosis, CCI score 1-year post-index, and primary treatment received.

Analyses were conducted in SAS 9.4 (SAS Institute, Cary, NC) and R studio (2022.07.1 Build 554).

## 3 Results

### 3.1 Patient characteristics

Overall, 2,180 patients with LA HNSCC met all criteria and were included in the study (**Figure 1**). Among them, 626 (28.7%) patients received primary surgery and were included in the resected cohort (median follow-up: 20.8 months), while 1,554 (71.3%) were included in the unresected cohort (median follow-up: 22.6 months) (**Table 1**). In the resected and unresected cohorts, respectively, the mean age at diagnosis was 74.5 (SD: 7.0) and 74.2 (6.7) years, 62.1% and 67.4% were male, and 81.0% and 82.0% were White. A majority of patients in both the resected (61.2%) and unresected (64.5%) cohorts had stage IVa disease at initial diagnosis. The most common tumor site at initial diagnosis was oral cavity (40.7%) in the resected cohort and oropharynx (38.8%) in the unresected cohort.

**Table 1:** Baseline characteristics of patients with LA HNSCC by primary treatment cohort^a^.

Patient characteristics	Overall	Resected LA HNSCC Cohort	Unresected LA HNSCC Cohort	*P*-value
	(N = 2,180)	(N = 626)	(N = 1,554)	
Demographic characteristics				
Age at diagnosis (years), mean ± SD	74.3 ± 6.8	74.5 ± 7.0	74.2 ± 6.7	0.361
Male, N (%)	1,436 (65.9)	389 (62.1)	1,047 (67.4)	0.020*
Race/ethnicity, N (%)				0.862
White	1,781 (81.7)	507 (81.0)	1,274 (82.0)	
Black	269 (12.3)	78 (12.5)	191 (12.3)	
Others/unknown	130 (6.0)	41 (6.5)	89 (5.7)	
Residential area at diagnosis, N (%)				0.097
Metropolitan	1,761 (80.8)	494 (78.9)	1,267 (81.5)	
Non-metropolitan/completely rural	419 (19.3)	132 (21.1)	287 (18.5)	
Region, N (%)				0.308
West	865 (39.7)	229 (36.6)	636 (40.9)	
South	655 (30.0)	200 (31.9)	455 (29.3)	
Northeast	387 (17.8)	116 (18.5)	271 (17.4)	
Midwest	273 (12.5)	81 (12.9)	192 (12.4)	
Year of initial diagnosis, N (%)				0.033*
2007	122 (5.6)	43 (6.9)	79 (5.1)	
2008	125 (5.7)	43 (6.9)	82 (5.3)	
2009	114 (5.2)	36 (5.8)	78 (5.0)	
2010	132 (6.1)	29 (4.6)	103 (6.6)	
2011	167 (7.7)	41 (6.5)	126 (8.1)	
2012	169 (7.8)	45 (7.2)	124 (8.0)	
2013	214 (9.8)	55 (8.8)	159 (10.2)	
2014	202 (9.3)	53 (8.5)	149 (9.6)	
2015	182 (8.3)	49 (7.8)	133 (8.6)	
2016	226 (10.4)	75 (12.0)	151 (9.7)	
2017	245 (11.2)	58 (9.3)	187 (12.0)	
2018	138 (6.3)	49 (7.8)	89 (5.7)	
2019	144 (6.6)	50 (8.0)	94 (6.0)	
Primary payer at initial diagnosis, N (%)				0.691
Medicare	1,624 (74.5)	470 (75.1)	1,154 (74.3)	
Others	556 (25.5)	156 (24.9)	400 (25.7)	
Clinical characteristics				
Disease stage group, N (%)				0.130
Stage III	787 (36.1)	240 (38.3)	547 (35.2)	
Stage IVa	1,173 (53.8)	325 (51.9)	848 (54.6)	
Stage IVb	160 (7.3)	50 (8.0)	110 (7.1)	
Stage IV unspecified/Missing stage^b^	60 (2.8)	11 (1.8)	49 (3.1)	
Tumor site at diagnosis, N (%)				<0.001*
Oropharynx	723 (33.2)	120 (19.2)	603 (38.8)	
Larynx	661 (30.3)	221 (35.3)	440 (28.3)	
Oral cavity	659 (30.2)	255 (40.7)	404 (26.0)	
Hypopharynx	137 (6.3)	30 (4.8)	107 (6.9)	
Tumor size at diagnosis (cm), mean ± SD	3.8 ± 4.2	3.7 ± 4.4	3.8 ± 4.1	0.167
CCI score, mean ± SD^c^	1.4 ± 1.6	1.5 ± 1.7	1.4 ± 1.6	0.286

CCI, Charlson Comorbidity Index; ICD-9/10, International Classification of Diseases, 9^th^/10^th^ edition; LA HNSCC, locoregionally advanced head and neck squamous cell carcinoma; SEER, Surveillance, Epidemiology, and End Results database; SD, standard deviation. *p<0.05.

a Sample sizes were suppressed for patient characteristics with N < 11.

b Missing stage included patients who had missing SEER stage group information.

c The conditions included in the CCI were identified using ICD-9/10 diagnosis codes reported by Quan et al. (2005) (28), excluding codes for cancer. The CCI weights were based on Quan et al. (2011) (29).

### 3.2 Primary treatment patterns

The majority (56.3%) of patients in the resected cohort received RT or RT with systemic therapy (ST) post-surgery, while 26.8% only received surgery (**Table 2**). The median (IQR) time from initial diagnosis to surgery was 14 (6,37) days while the median time from surgery to post-surgery treatment was 21 (13,32) days. Among patients who received surgery followed by RT and ST, 66.2% received platinum-based ST (cisplatin-based: 80.2%; carboplatin-based: 19.8%) and 33.8% received cetuximab. Older patients at index and patients with larynx or oral cavity cancer (vs. those with oropharynx cancer) were less likely to receive post-operative RT/ST (**Supplementary Table 1**).

**Table 2:** Distribution of primary treatment patterns^a^.

Primary treatment patterns	N	Percentage
**Resected**	**626**	**100.0%**
Surgery only^b^	168	26.8%
Systemic/radiation therapy + surgery^c^	85	13.6%
Surgery + radiation therapy^d^	207	33.1%
Surgery + systemic and radiation therapy^d^	145	23.2%
Surgery + systemic therapy^d^	<11	<1.8%
Systemic/radiation therapy + surgery + systemic/radiation therapy^e^	>10	>1.6%
**Unresected**	**1,554**	**100.0%**
Systemic therapy only^f^	<11	<0.7%
Radiation therapy only^g^	>534	>34.4%
Systemic therapy + radiation therapy	1,009	64.9%
Cisplatin-based	517	33.3%
Carboplatin-based	163	10.5%
Non-platinum-based	329	21.2%

a Treatment categories with N<11 were not reported.

b Patients received surgery within 4 months after the initial diagnosis and did not receive other treatments between initial diagnosis and surgery, and within 60 days after surgery.

c Patients received surgery within 4 months after the initial diagnosis and did not receive other primary treatment between initial diagnosis and surgery, and received and completed at least one of the radio- or systemic therapies any time after the initial diagnosis but before the surgery. Patients did not receive any radio- or systemic therapy within 60 days after surgery.

d Patients received surgery within 4 months after the initial diagnosis and did not receive any treatment between initial diagnosis and surgery, and received at least one of the radio- or systemic therapies within 60 days after the initial surgery.

e Patients received surgery within 4 months after the initial diagnosis and did not receive other primary treatment between initial diagnosis and surgery; received and completed at least one of radio- or systemic therapies any time after the initial diagnosis but before the surgery; received at least one of the radio- or systemic therapies within 60 days after the initial surgery.

f Patients had at least one claim with systemic therapy within 4 months after the initial diagnosis, and all unique agents received within the first 30 days following initiation of the first systemic therapy were considered as part of the primary treatment. Patients did not initiate radiation therapy during systemic therapy or within 60 days after the last administration of systemic therapy.

g Patients had at least one claim with radiation therapy within 4 months after the initial diagnosis and did not initiate any systemic therapy before the completion of radiation therapy.

In the unresected cohort, 64.9% of patients received definitive ST with RT (>98% concurrently), while >34.4% received RT only (**Table 2**). Among patients who received definitive ST with RT, 67.4% received platinum-based ST and 32.6% received non-platinum-based ST. Among patients on concurrent ST with RT, 67.5% received platinum-based ST (cisplatin-based: 76.0%; carboplatin-based: 24.0%), and 30.6% received cetuximab. Older patients at index, patients with higher CCI score at baseline, and patients with larynx and oral cavity cancer (vs. those with oropharynx cancer) were less likely to receive definitive ST with RT (**Supplementary Table 2**).

### 3.3 rwEFS and factors associated with rwEFS

**Figure 2:**
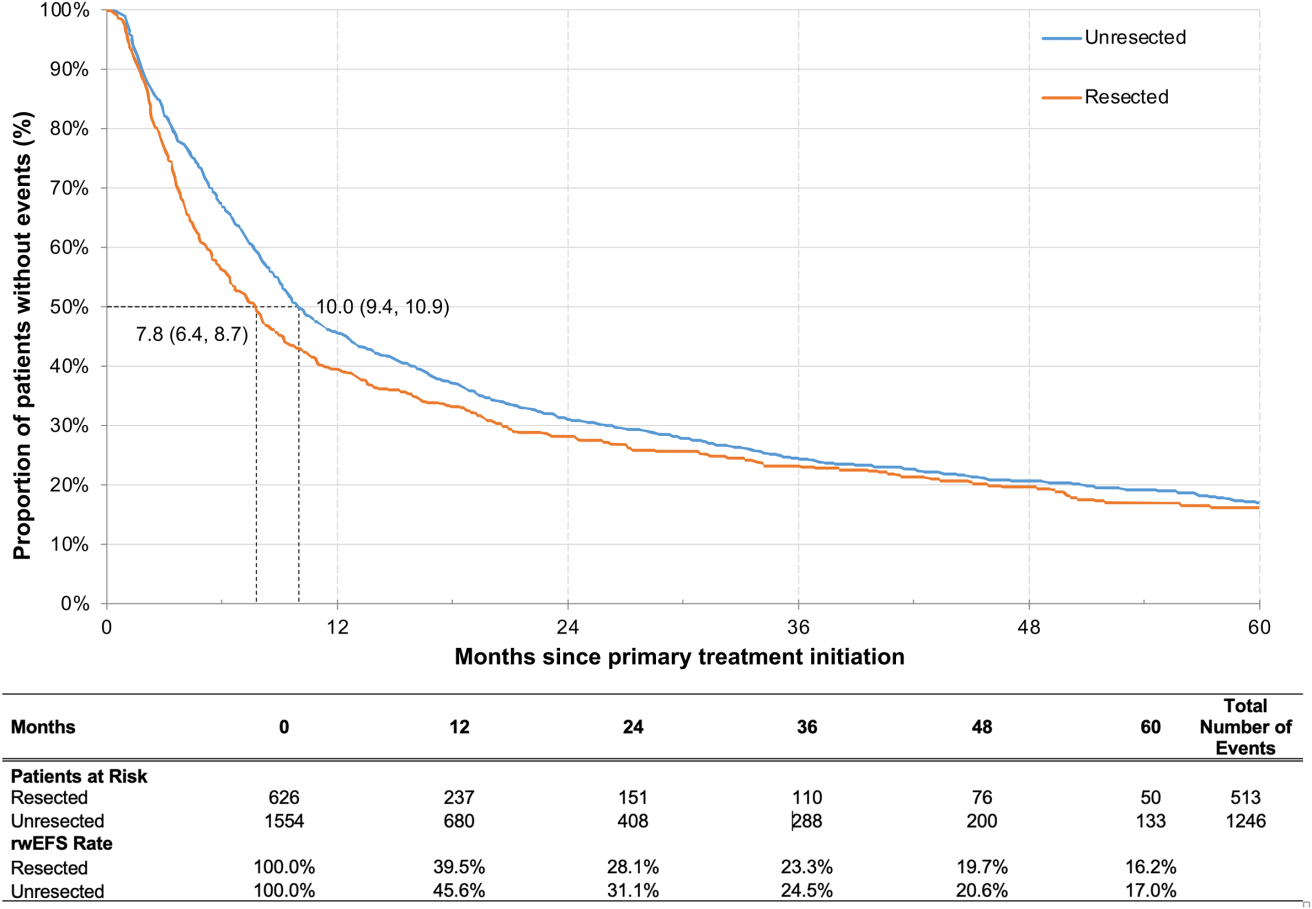
Kaplan-Meier analysis of rwEFS by general treatment pattern among patients with resected or unresected LA HNSCC. LA HNSCC, locoregionally advanced head and neck squamous cell carcinoma; rwEFS, real-world event-free survival.

The median rwEFS was 7.8 (95% CI: 6.4, 8.7) months for the resected cohort and 10.0 (95% CI: 9.4, 10.9) months for the unresected cohort, with a 5-year rwEFS rate of 16.2% and 17.0%, respectively (**Figure 2**). When stratifying by tumor sites, patients with oropharynx cancer had the highest 5-year rwEFS rate in both the resected (19.3%) and unresected cohorts (20.6%), while the lowest 5-year rwEFS rate was observed for patients with hypopharynx cancer in resected cohort (9.3%) and for patients with oral cavity cancer in the unresected cohort (11.1%) (**Supplementary Figures 1, 2**). In the sensitivity analysis limiting EFS events to only initiation of the next line of therapy and death, the median rwEFS was extended to 8.8 months for the resected cohort and 12.9 months for the unresected cohort (**Supplementary Figure 3**).

Among patients with resected tumors, the adjusted Cox model indicated that, compared to surgery alone, RT with or without ST before surgery (HR [95% CI]: 0.70 [0.52, 0.95]), RT after surgery (0.63 [0.50, 0.80]), and RT with ST after surgery (0.50 [0.38, 0.67]) were all associated with significantly longer rwEFS (all *p*<0.05) (**Table 3**). Additionally, stage IVa (HR [95% CI]: 1.28 [1.05, 1.55]) or IVb (2.07 [1.47, 2.92]) as compared to stage III at diagnosis and higher baseline CCI score (1.06 [1.00, 1.12]) were associated with significantly shorter rwEFS (all *p*<0.05).

**Table 3:** Multivariable Cox proportional hazard models between primary treatment and rwEFS and rwOS among patients with resected LA HNSCC^a^.

Covariates	rwEFS		rwOS	
	Hazard ratio (95% CI)	*P*-value	Hazard ratio (95% CI)	*P*-value
Primary treatment				
Surgery only	*Reference*		*Reference*	
Systemic/radiation therapy + surgery	0.70 (0.52, 0.95)	0.023*	0.85 (0.61, 1.20)	0.354
Surgery + radiation therapy	0.63 (0.50, 0.80)	<0.001*	0.73 (0.57, 0.95)	0.020*
Surgery + systemic and radiation therapy	0.50 (0.38, 0.67)	<0.001*	0.50 (0.36, 0.70)	<0.001*
Age at index date	1.01 (0.99, 1.02)	0.404	1.05 (1.04, 1.07)	<0.001*
Sex				
Male	*Reference*		*Reference*	
Female	0.93 (0.76, 1.14)	0.498	0.85 (0.67, 1.07)	0.174
Race				
White	*Reference*		*Reference*	
Black	1.10 (0.83, 1.47)	0.507	1.00 (0.72, 1.39)	0.986
Other	0.97 (0.66, 1.42)	0.867	0.96 (0.62, 1.49)	0.866
Region				
West	*Reference*		*Reference*	
Mid-west	0.83 (0.62, 1.11)	0.203	0.90 (0.64, 1.28)	0.566
Northeast	0.80 (0.61, 1.04)	0.091	0.89 (0.65, 1.21)	0.442
South	0.90 (0.72, 1.12)	0.338	1.22 (0.94, 1.58)	0.129
Stage				
Stage III	*Reference*		*Reference*	
Stage IVa	1.28 (1.05, 1.55)	0.013*	1.30 (1.03, 1.63)	0.025*
Stage IVb	2.07 (1.47, 2.92)	<0.001*	1.92 (1.31, 2.80)	<0.001*
Stage IV unspecified	0.70 (0.31, 1.61)	0.404	0.51 (0.18, 1.38)	0.184
Site				
Oropharynx	*Reference*		*Reference*	
Hypopharynx	1.24 (0.79, 1.93)	0.351	2.01 (1.23, 3.28)	0.005*
Larynx	1.06 (0.81, 1.39)	0.654	1.16 (0.85, 1.59)	0.345
Oral cavity	0.88 (0.67, 1.17)	0.379	0.90 (0.65, 1.26)	0.548
CCI score	1.06 (1.00, 1.12)	0.049*	1.14 (1.08, 1.21)	<0.001*

CCI, Charlson Comorbidity Index; CI, confidence interval; LA HNSCC, locoregionally advanced head and neck squamous cell carcinoma; rwEFS, real-world event-free survival; rwOS, real-world overall survival. *p<0.05.

a Primary treatment class, race, and disease stage with small number of patients (N<11) were excluded from this adjusted analysis.

Among patients with unresected tumors, patients receiving ST with RT had significantly longer rwEFS than patients receiving RT only (HR [95% CI: 0.80 [0.70, 0.91]; *p*<0.001) (**Table 4**). Conversely, older age at index (HR [95% CI: 1.03 [1.02, 1.04]), stage IVa (1.39 [1.23, 1.58]) or IVb (1.98 [1.56, 2.51]) vs. stage III disease at diagnosis, hypopharynx vs. oropharynx cancer site (1.32 [1.05, 1.66]), and higher CCI score (1.12 [1.08, 1.16]) were associated with significantly shorter rwEFS (all *p*<0.05).

**Table 4:** Multivariable Cox proportional hazard model between primary treatment and rwEFS and rwOS among patients with unresected LA HNSCC^a^.

Covariates	rwEFS		rwOS	
	Hazard ratio (95% CI)	*P*-value	Hazard ratio (95% CI)	*P*-value
Primary treatment				
Radiation therapy only	*Reference*		*Reference*	
Systemic + radiation therapy	0.80 (0.70, 0.91)	<0.001*	0.70 (0.61, 0.81)	<0.001*
Age at index date	1.03 (1.02, 1.04)	<0.001*	1.06 (1.05, 1.07)	<0.001*
Sex				
Male	*Reference*		*Reference*	
Female	0.93 (0.82, 1.06)	0.269	0.95 (0.82, 1.10)	0.504
Race				
White	*Reference*		*Reference*	
Black	0.96 (0.80, 1.14)	0.617	1.09 (0.89, 1.32)	0.414
Other	0.73 (0.56, 0.95)	0.021*	0.77 (0.57, 1.04)	0.087
Region				
West	*Reference*		*Reference*	
Mid-west	1.07 (0.89, 1.28)	0.486	1.17 (0.96, 1.44)	0.124
Northeast	0.91 (0.77, 1.07)	0.234	0.99 (0.82, 1.19)	0.908
South	0.91 (0.79, 1.05)	0.207	1.12 (0.95, 1.31)	0.183
Stage				
Stage III	*Reference*		*Reference*	
Stage IVa	1.39 (1.23, 1.58)	<0.001*	1.38 (1.20, 1.60)	<0.001*
Stage IVb	1.98 (1.56, 2.51)	<0.001*	2.91 (2.26, 3.75)	<0.001*
Stage IV unspecified	1.42 (0.99, 2.01)	0.054	1.60 (1.09, 2.35)	0.017*
Site				
Oropharynx	*Reference*		*Reference*	
Hypopharynx	1.32 (1.05, 1.66)	0.017*	1.43 (1.11, 1.84)	0.006*
Larynx	1.02 (0.89, 1.19)	0.744	1.13 (0.95, 1.33)	0.166
Oral Cavity	1.08 (0.92, 1.27)	0.325	1.24 (1.03, 1.49)	0.024*
CCI Score	1.12 (1.08, 1.16)	<0.001*	1.18 (1.14, 1.22)	<0.001*

CCI, Charlson Comorbidity Index; CI, confidence interval; LA HNSCC, locoregionally advanced head and neck squamous cell carcinoma; rwEFS, real-world event-free survival; rwOS, real-world overall survival. *p<0.05.

a Primary treatment class, race, and disease stage with small number of patients (N<11) were excluded from this adjusted analysis.

### 3.4 rwOS and factors associated with rwOS

**Figure 3:**
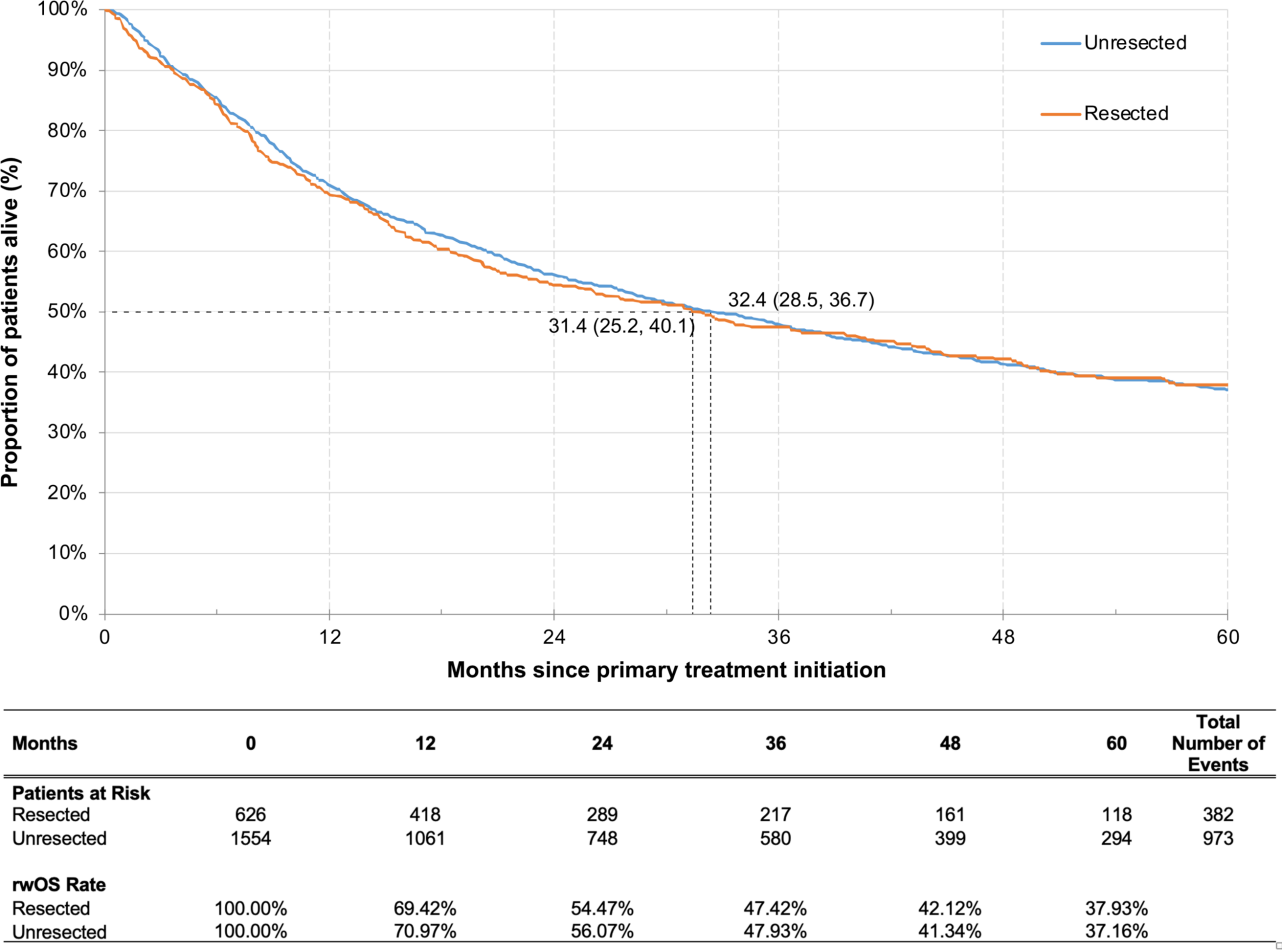
Kaplan-Meier analysis of rwOS by general treatment pattern among patients with resected or unresected LA HNSCC. LA HNSCC, locally advanced head and neck squamous cell carcinoma; rwOS, real-world overall survival.

The median rwOS was 31.4 (95% CI: 25.2, 40.1) months for the resected cohort and 32.4 (28.5, 36.7) months for the unresected cohort; the 5-year rwOS rates were 37.9% and 37.2%, respectively (**Figure 3**). Similar to rwEFS, when stratifying by tumor sites, patients with oropharynx cancer had the highest 5-year rwOS rate in both the resected (52.0%) and unresected cohorts (43.2%), while the lowest 5-year rwOS rate was observed for patients with hypopharynx cancer in resected cohort (18.4%) and for patients with oral cavity cancer in unresected cohort (28.8%) (**Supplementary Figures 4, 5**).

The adjusted Cox model indicated that post-surgery RT (HR [95% CI]: 0.73 [0.57, 0.95]) or RT with ST (0.50 [0.36, 0.70]) vs. only surgery in the resected cohort (**Table 3**), and definitive ST with RT (0.70 [0.61, 0.81]) vs. RT only in the unresected cohort (**Table 4**), were associated with significantly longer rwOS (all *p*<0.05). For both the resected and unresected cohorts, older age at the index date, higher tumor stage at diagnosis, hypopharynx cancer site (vs. oropharynx), and higher CCI score were all associated with significantly shorter rwOS (all *p*<0.05). Additionally, cancer site of oral cavity (HR [95% CI]: 1.24 [1.03, 1.49]; *p*<0.05) was associated with significantly shorter rwOS compared to oropharynx in the unresected cohort (**Table 4**).

### 3.5 Cumulative incidence rate of recurrence

The 5-year cumulative incidence rate of recurrence was 56.1% in the resected cohort and 54.1% in the unresected cohort (**Supplementary Figures 6, 7**). Most of the recurrence events (75% in the resected cohort and 67% in the unresected cohort) occurred within the first year after primary treatment initiation. When stratifying by tumor site, patients with oropharynx cancer had the highest cumulative incidence rate of recurrence in the resected cohort (**Supplementary Figure 8**). In the unresected cohort, the cumulative incidence rate of recurrence was similar across tumor sites (**Supplementary Figure 9**).

### 3.6 Correlation between rwEFS and rwOS and landmark analysis

The estimated normal score rank correlation demonstrated a statistically significant positive correlation between rwEFS and rwOS for both the resected (*r* [95% CI]: 0.69 [0.63, 0.73]) and unresected (0.68 [0.63, 0.73]) cohorts (both *p*<0.001).

**Figure 4:**
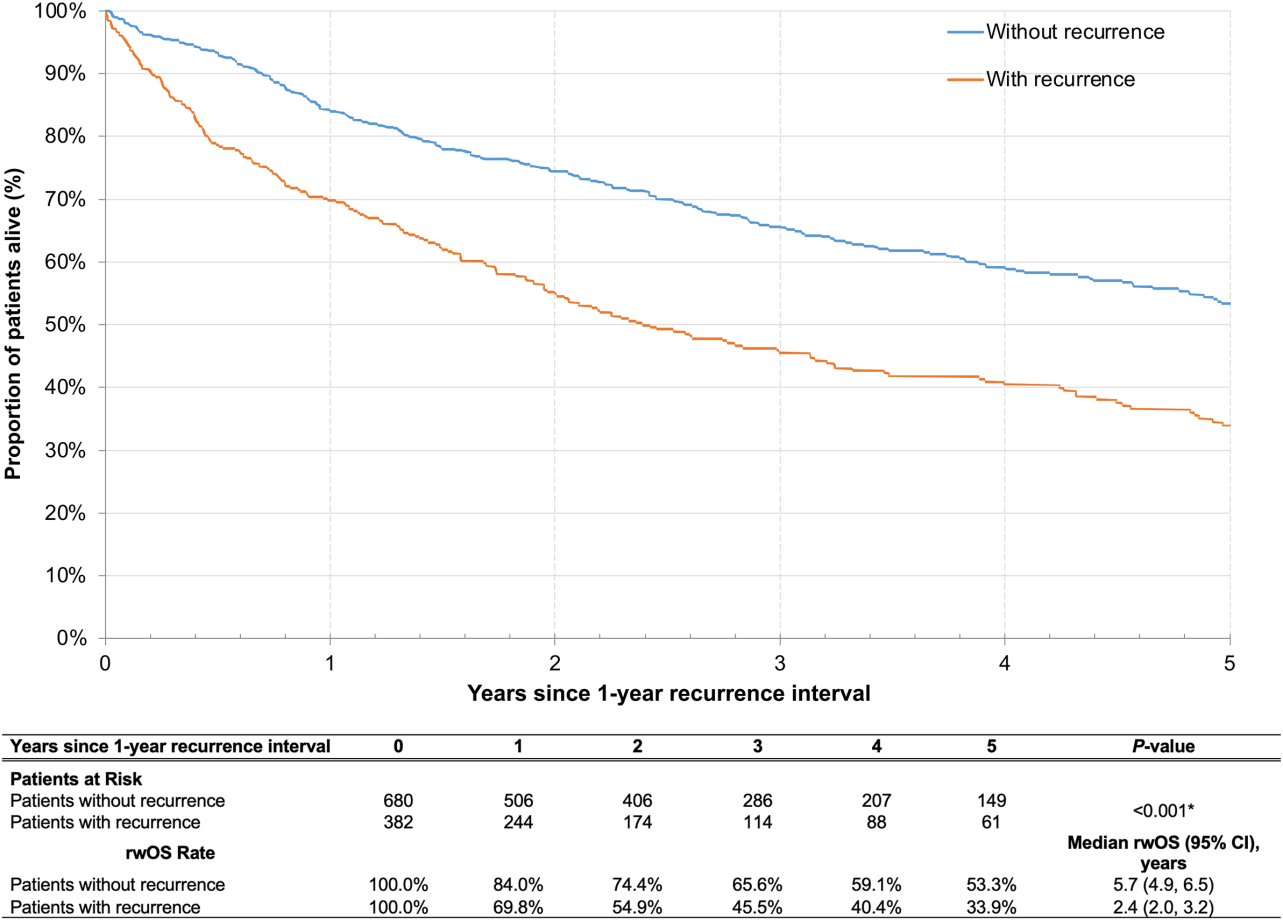
Kaplan-Meier analysis of rwOS comparing patients with unresected LA HNSCC who did and did not have a recurrence event within 1 year following primary treatment initiation. CI, confidence interval; LA HNSCC, locoregionally advanced head and neck squamous cell carcinoma; rwOS, real-world overall survival. *p<0.05.

**Figure 5:**
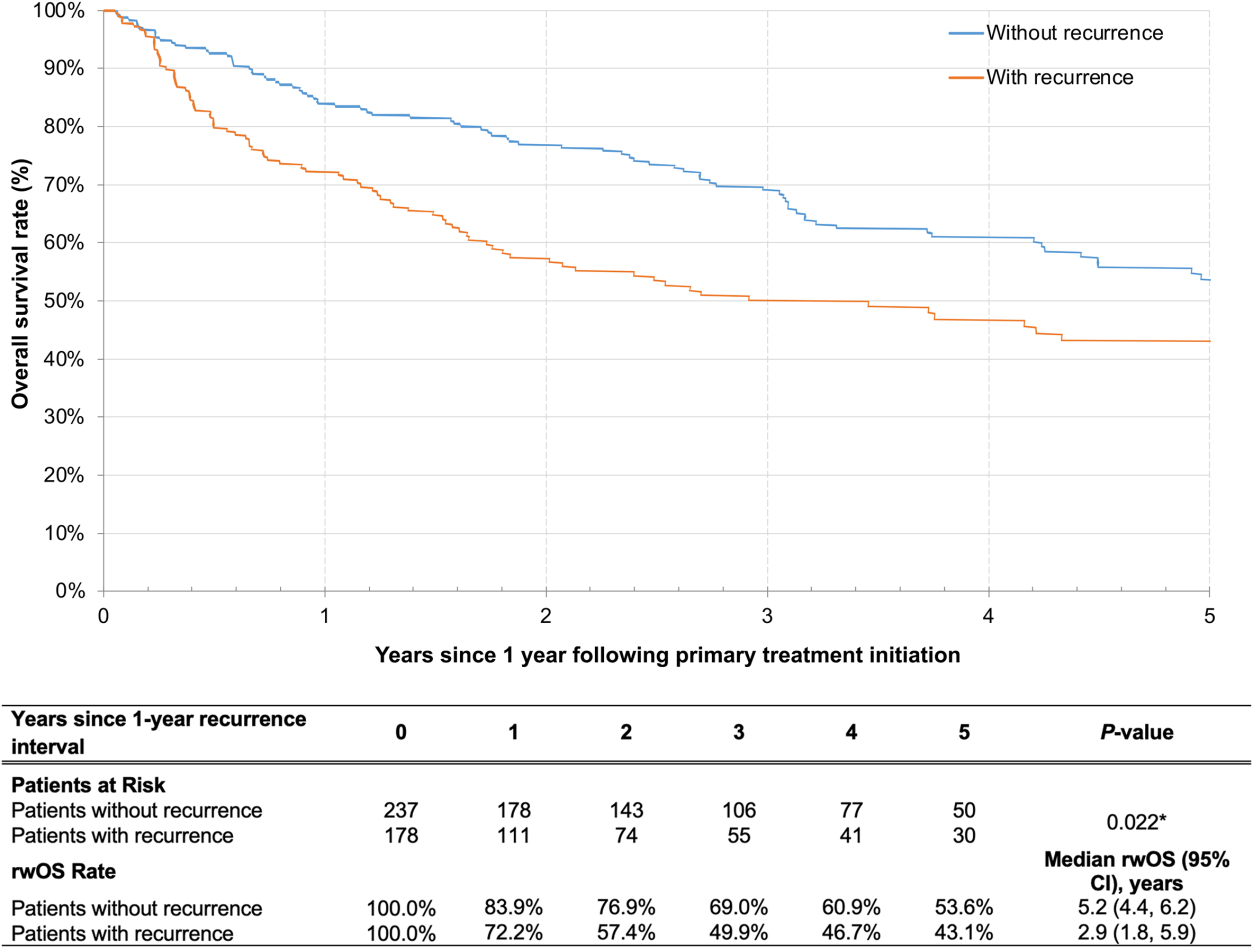
Kaplan-Meier analysis of rwOS comparing patients with resected LA HNSCC who did and did not have a recurrence event within 1 year following primary treatment initiation. CI, confidence interval; LA HNSCC, locoregionally advanced head and neck squamous cell carcinoma; rwOS, real-world overall survival. *p<0.05.

For the unresected cohort, patients with recurrence within the first year of primary treatment initiation had significantly shorter subsequent OS than those without recurrence (**Table 5**, **Figure 4**). Specifically, median rwOS since 1-year post-index landmark was 2.4 years for patients with recurrence within the first year of primary treatment initiation as compared to 5.7 years for patients without recurrence in the same period (*p*<0.001). The adjusted Cox models indicated that patients with unresected disease had a significant 91% increased risk of death associated with recurrence within the first year of primary treatment initiation as compared to those without recurrence in the same period. Similarly, in the resected cohort, the median rwOS since the landmark was 2.9 years and 5.2 years for patients with and without recurrence, respectively, in the first year after primary treatment initiation (*p*=0.02). After adjusting for covariates, patients in the resected cohort who experienced recurrence within the first year after primary treatment had a 31% increased risk of death compared with those who did not have recurrence, although the difference was not statistically significant (*p*=0.092) (**Table 5**, **Figure 5**).

**Table 5:** Cox proportional hazards model of rwOS for patients with versus without recurrence within 1 year after primary treatment.

Cohort	Median rwOS after 1-year recurrence interval, years		Adjusted hazard ratio (95% CI)^a,b^	*P-*value
	With recurrence	Without recurrence		
Resected	2.9	5.2	1.31 (0.96, 1.80)	0.092
Unresected	2.4	5.7	1.91 (1.60, 2.29)	<0.001*

CI, confidence interval; rwOS, real-world overall survival. *p<0.05.

a Adjusted variables included primary treatment, age at index date, sex, race, region, disease stage, tumor site, and Charlson Comorbidity Index score.

b Primary treatment class, race, and disease stage with N<11 were excluded from this adjusted analysis.

## 4 Discussion

This retrospective observational study described the real-world treatment patterns, rwEFS, rwOS, cumulative incidence rate of recurrence, and correlations between rwEFS and rwOS among elderly patients with resected and unresected LA HNSCC in the US. The results indicated that 56% of patients with resected HNSCC received RT and/or ST after surgery, while 65% of patients not undergoing resection received definitive ST with RT. The median rwEFS and rwOS were 7.8 and 31.4 months, respectively, in the resected cohort and 10.0 and 32.4 months in the unresected cohort. Additionally, a significant positive correlation between rwEFS and rwOS was observed in both the resected and unresected cohorts.

This study summarized the real-world treatment patterns of LA HNSCC and found that 28.7% of patients underwent resection as primary treatment, and among them, over half received post-operative RT with/without ST, the current standard treatment approach. Most patients who did not undergo resection received definitive RT and ST. These findings are consistent with those of a prior study by Hansen et al., also using SEER-Medicare data (1991-2011), which reported that 30% of elderly patients (mean age: 75 years) with stage III HNSCC at initial diagnosis, and 38% with stage IV, underwent resection (20). Among those resected, approximately 66% received post-operative RT and/or ST, while 55% of patients with unresected tumors received definitive RT and ST. Additionally, a US chart review study of 338 patients with newly diagnosed oropharyngeal and laryngeal SCC (2000-2012), with a median age of 61 years at diagnosis, reported that 22% received surgery as primary treatment (21). The lower proportion of patients undergoing resection in the chart review study compared to that observed in this study may be related to the differing cancer sites, human papillomavirus (HPV) status, or the use of trans-oral robotic resection (data on the latter two are not available in SEER-Medicare data). Specifically, the chart review study did not include oral cavity cancer, which was the most resectable cancer site of the four sites included in this study. When restricting our population to patients with oropharyngeal and laryngeal cancers, the percentage of resected tumors was similar between studies.

For both the resected and unresected cohorts, the rwOS observed in this study was generally consistent with the findings of prior studies. A recent study by Saba et al. among elderly patients diagnosed with LA HNSCC between 2014–2017 using linked SEER-Medicare data evaluated rwOS by cancer site in resected and unresected patients (22). They reported a median rwOS in the unresected cohort of not reached, 34.6 months, 34.1 months, and 15.3 months for oropharynx, larynx, hypopharynx, and oral cavity cancer, respectively, and a median rwOS in the resected cohort of not reached, 30.6 months, and 30.0 months for oropharynx, oral cavity, and larynx cancer, respectively (22). Similarly, another study by Rühle et al. reported a median OS of 36 months among patients aged ≥65 years with LA HNSCC of the oral cavity, oropharynx, hypopharynx, or larynx undergoing non-surgical primary treatments between 2005–2019 at 12 academic centers in the US and Europe (23).

Few studies have reported the rwEFS for patients with LA HNSCC. However, Rühle et al. reported a median progression-free survival (PFS), including events for death, local or locoregional progression, and development of distant metastases, of 20 months for patients with LA HNSCCs of the oral cavity, oropharynx, hypopharynx, or larynx undergoing definitive RT, alone or with simultaneous ST (23). Additionally, a retrospective cohort study of patients with LA HNSCC diagnosed 2015–2018 in England, including patients with and without primary surgery, reported a median EFS of 12.3 months (6). A comparatively shorter EFS was observed among the Medicare patients in the current study, potentially due to their generally older age distribution, which is associated with worse outcomes in this patient population. Because the diagnosis codes of secondary malignancy can be used for diagnostic medical procedures, they can be observed from patients with suspicious but not confirmed recurrent disease. Therefore, using it as an indicator for recurrent disease may lead to an underestimation of EFS. Indeed, a comparable but longer median rwEFS was observed in the sensitivity analysis limiting EFS events to only initiation of the next line of therapy and death. Finally, the slightly numerically longer median rwEFS observed for the unresected compared to the resected cohort may be due to the differing distribution of tumor sites. For example, the unresected cohort had a higher proportion with oropharynx cancer than the resected cohort, which had longer EFS. Furthermore, patients were not randomized to receive surgical vs. non-surgical treatments. The underlying factors influencing treatment decision making, such as frailty, were not observed. However, this study did not intend to compare outcomes of the two cohorts.

In the resected cohort, after controlling for other baseline demographic and clinical characteristics, the use of pre-operative RT with or without ST and post-operative RT or RT with ST (vs surgery only) was associated with significantly longer rwEFS. Additionally, the use of post-operative RT or RT with ST (vs surgery only) were also associated with significantly longer rwOS in the resected cohort. Similarly, the adjusted analyses in the unresected cohort indicated that definitive ST and RT (vs. RT only) was associated with significantly longer rwEFS and rwOS. These findings are generally consistent with those of previous studies (4, 24), but should be interpreted with caution because some important prognosis factors, such as HPV status, Eastern Cooperative Oncology Group (ECOG) performance status, and smoking history, were not available in the data.

To our knowledge, this is the first study to assess the correlation between rwEFS and rwOS in patients with LA HNSCC. The results demonstrated a significant positive correlation between rwEFS and rwOS among both the resected and unresected cohorts. Further, patients who experienced disease recurrence, an important adverse clinical event, within the first year of treatment initiation had significantly shorter subsequent rwOS than those without recurrence within the same period. Due to the limited sample size, particularly in the resected cohort, landmark analyses beyond 1 year after primary treatment initiation were not conducted in the current study. Nevertheless, the present findings are consistent with those of a meta-analysis using data from 31 randomized controlled trials among patients who were newly diagnosed with LA HNSCC and received definitive CRT, which reported a strong association between the two outcomes of EFS and OS (11).

### 4.1 Limitations

The results of this study are subject to several limitations, including those common among retrospective claims database studies. First, we only included elderly patients because the patient population in the linked SEER-Medicare database are all Medicare beneficiaries (i.e., generally eligible for coverage at age 65 years). Therefore, the results from this study may not be generalizable to a younger patient population, those with another form of insurance, or the uninsured. Second, due to the nature of administrative claims data, HNSCC recurrence cannot be identified directly; therefore, an algorithm that relied on various procedure codes, diagnosis codes, drug codes, and clinician opinions was used (25–27). Coding inaccuracies may have led to misclassification bias (e.g., treatment misclassification) and misidentification of patients with HNSCC recurrence. Future studies that incorporate detailed medical-record review will be essential to validate our findings. Third, some important prognostic factors, such as HPV status, ECOG performance status, alcohol use, and smoking history, were not available in the data. The results may also be confounded by these variables. Therefore, future studies using a database with this information may be warranted to confirm the present results.

## 5 Conclusions

This retrospective study of elderly patients with LA HNSCC provides important insights into the real-world clinical outcomes of patients who did or did not receive surgical resection. The results underscore the impact of treatment strategies and patient characteristics on rwEFS and rwOS and highlight the unmet need for novel treatments in patients with LA HNSCC. Notably, rwEFS and rwOS were found to have a significant positive association among both the resected and unresected cohorts, supporting the use of rwEFS as a predictor of OS to facilitate clinical trials assessing novel treatments for LA HNSCC. Additionally, disease recurrence within the first year after treatment initiation was associated with lower OS in both the resected and unresected cohorts, a relationship which was statistically significant in the unresected cohort. Further clinical research is needed to refine treatment strategies and explore innovative approaches to delay recurrence and improve long-term survival in this challenging disease.

## Data Availability

The datasets presented in this article are not readily available because The study used the SEER-Medicare Linked Database to generate the data. The SEER-Medicare Linked Database is not publicly available due to the sensitive nature of the data and risk of reidentification. Investigators are required to obtain approval from SEER-Medicare to obtain the data. Requests to access the datasets should be directed to https://healthcaredelivery.cancer.gov/seermedicare/obtain/requests.html.
