# Correction: Real-world treatment patterns and clinical outcomes among elderly patients with locoregionally advanced head and neck squamous cell carcinoma in the United States

**DOI:** 10.3389/fonc.2026.1805179

**Published:** 2026-02-17

**Authors:** Dandan Zheng, Su Zhang, Behzad Bidadi, Nati Lerman, Yan Song, Rui Song, Jiayang Li, Anyu Zhu, Yuexin Tang, James Signorovitch, Sanjay Merchant, Glenn J. Hanna

**Affiliations:** 1 Outcomes Research, Merck & Co., Inc., Rahway, NJ, United States; 2 Analysis Group, Inc., Boston, MA, United States; 3 Oncology Late Stage Development, Merck & Co., Inc., Rahway, NJ, United States; 4 Center for Head and Neck Oncology, Dana-Farber Cancer Institute, Boston, MA, United States

**Keywords:** event-free survival, head and neck squamous cell carcinoma, locoregionally advanced, medicare, overall survival, real world, treatment patterns

There was a mistake in **Figure 1** as published. In the patient selection flow chart, the inequality symbols (≥) were incorrectly displayed as question marks (?), which affected the inclusion-criteria text (e.g., “Age ≥ 66 years” and the continuous enrollment criteria). The corrected **Figure 1** appears below.

**Figure 1:**
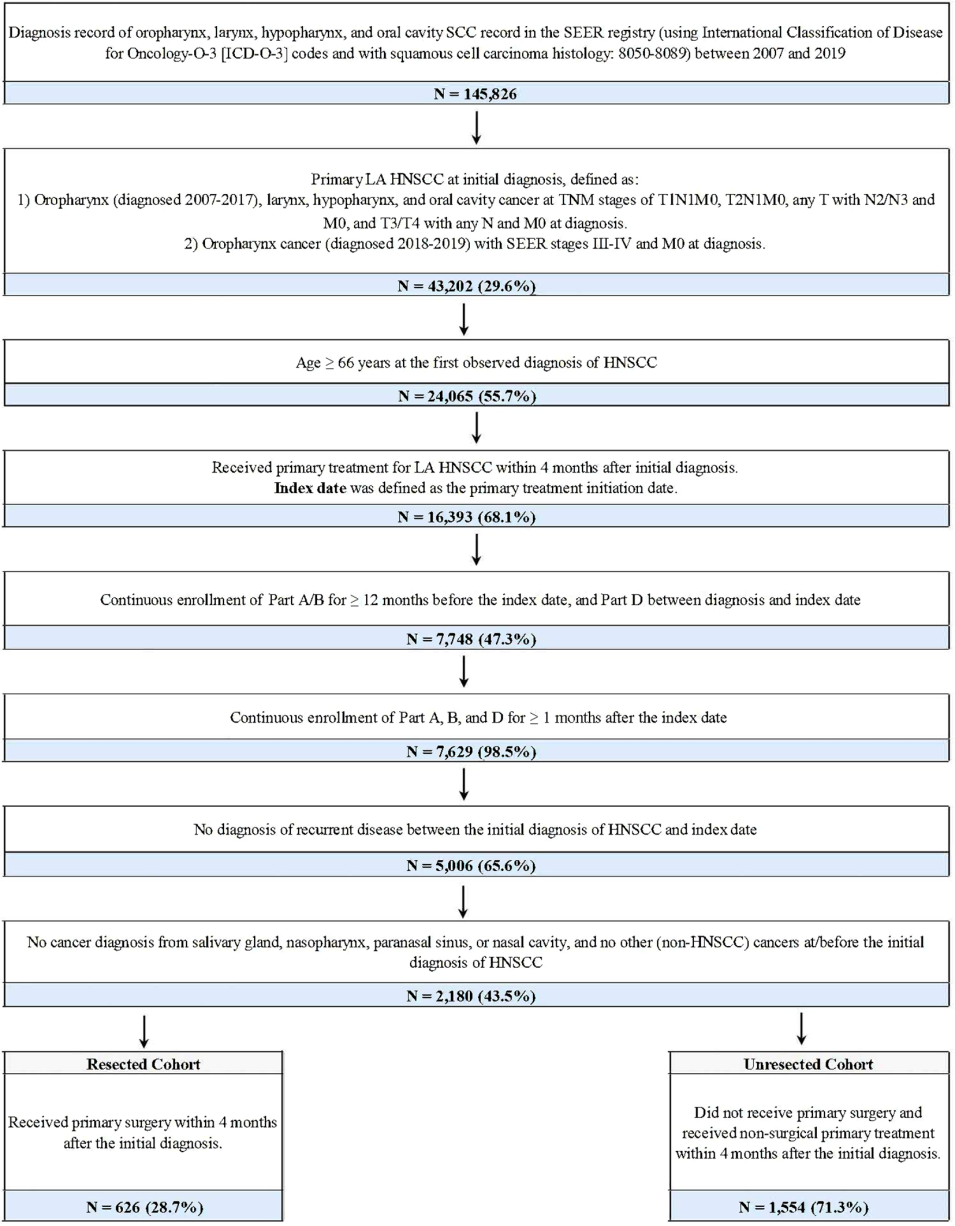
Sample selection of LA HNSCC patients.

The original version of this article has been updated.

