# Supplementary material for: Real-world treatment patterns and clinical outcomes among elderly patients with locoregionally advanced head and neck squamous cell carcinoma in the United States
Source: Front Oncol. 2025 Sep 10;15:1606990. doi: 10.3389/fonc.2025.1606990 (PMC12457382; doi:10.3389/fonc.2025.1606990)
Supplement: Supplementary file 1 [file Table1.docx]

**SUPPLEMENTARY MATERIAL *for “*Real-world Treatment Patterns and Clinical Outcomes among Elderly Patients with Locoregionally Advanced Head and Neck Squamous Cell Carcinoma in the United States”**

**Supplementary Table 1.** **Factors associated with treatment modalities among patients with resected LA HNSCC^a^**

| **Surgery + adjuvant therapy vs. Surgery only** | **Odds ratio (95% CI)** | ***P*-value** |
| --- | --- | --- |
|  |  |  |
| Age at Index Date | 0.94 (0.91, 0.97) | <0.001* |
| Sex |  |  |
| Male | *Reference* |  |
| Female | 0.80 (0.51, 1.24) | 0.316 |
| Race |  |  |
| White | *Reference* |  |
| Black | 0.85 (0.46, 1.58) | 0.602 |
| Other | 0.99 (0.44, 2.22) | 0.980 |
| Region |  |  |
| West | *Reference* |  |
| Mid-west | 1.13 (0.56, 2.26) | 0.735 |
| Northeast | 0.84 (0.47, 1.50) | 0.555 |
| South | 1.13 (0.67, 1.93) | 0.645 |
| Residential area |  |  |
| Metropolitan area | *Reference* |  |
| Non-metropolitan area | 0.58 (0.34, 1.01) | 0.053 |
| Stage |  |  |
| Stage III | *Reference* |  |
| Stage IVa | 0.80 (0.52, 1.22) | 0.299 |
| Stage IVb | 0.77 (0.34, 1.75) | 0.532 |
| Stage IV unspecified | 0.22 (0.04, 1.34) | 0.101 |
| Site |  |  |
| Oropharynx | *Reference* |  |
| Hypopharynx | 1.28 (0.33, 4.94) | 0.717 |
| Larynx | 0.34 (0.18, 0.66) | 0.001* |
| Oral Cavity | 0.19 (0.10, 0.37) | <0.001* |
| CCI Score | 0.89 (0.79, 1.00) | 0.053 |

**Abbreviations:** CCI, Charlson Comorbidity Index; CI, confidence interval**;** LA HNSCC, locoregionally advanced head and neck squamous cell carcinoma. **p*<0.05.

**Notes**: ^a^ Race and disease stage with small number of patients (N<11) were excluded from this adjusted analysis.

**Supplementary Table 2.** **Factors associated with treatment modalities among patients with unresected LA HNSCC^a^**

| **ST + RT vs. RT only** | **Odds ratio (95% CI)** | ***P*-value** |
| --- | --- | --- |
|  |  |  |
| Age at index date | 0.93 (0.92, 0.95) | <0.001* |
| Sex |  |  |
| Male | *Reference* |  |
| Female | 0.81 (0.63, 1.05) | 0.110 |
| Race |  |  |
| White | *Reference* |  |
| Black | 0.76 (0.53, 1.08) | 0.126 |
| Other | 0.63 (0.38, 1.03) | 0.067 |
| Region |  |  |
| West | *Reference* |  |
| Mid-west | 0.77 (0.53, 1.12) | 0.177 |
| Northeast | 0.90 (0.65, 1.26) | 0.548 |
| South | 1.01 (0.75, 1.36) | 0.938 |
| Residential area |  |  |
| Metropolitan area | *Reference* |  |
| Non-metropolitan area | 0.94 (0.69, 1.29) | 0.715 |
| Stage |  |  |
| Stage III | *Reference* |  |
| Stage IVa | 1.20 (0.94, 1.55) | 0.147 |
| Stage IVb | 1.03 (0.63, 1.66) | 0.918 |
| Stage IV unspecified | 1.57 (0.72, 3.43) | 0.259 |
| Site |  |  |
| Oropharynx | *Reference* |  |
| Hypopharynx | 0.80 (0.49, 1.29) | 0.353 |
| Larynx | 0.66 (0.49, 0.90) | 0.008* |
| Oral cavity | 0.26 (0.19, 0.35) | <0.001* |
| CCI score | 0.93 (0.87, 1.00) | 0.049* |

**Abbreviations:** CCI, Charlson Comorbidity Index; CI, confidence interval; LA HNSCC, locoregionally advanced head and neck squamous cell carcinoma; RT, radiotherapy; ST, systemic therapy. **p*<0.05.

**Notes**: ^a^ Race and disease stage with small number of patients (N<11) were excluded from this adjusted analysis.

**Supplementary Figure 1. Kaplan-Meier analysis of rwEFS by cancer site among patients with resected LA HNSCC**

|  | **Months since primary treatment initiation** | | | | | | | | | | | |  | |
| --- | --- | --- | --- | --- | --- | --- | --- | --- | --- | --- | --- | --- | --- | --- |
|  |  |  |  |  |  |  |  |  |  |  |  |  | |  |
| **Months** | **0** |  | **12** |  | **24** |  | **36** |  | **48** |  | **60** | **Total Number of Events** | |  |
| **Patients at Risk** |  |  |  |  |  |  |  |  |  |  |  |  | |  |
| Hypopharynx | 30 |  | n* |  | n* |  | n* |  | n* |  | n* | n* | |  |
| Larynx | 221 |  | 91 |  | 46 |  | 29 |  | n* |  | n* | 183 | |  |
| Oral cavity | 255 |  | 89 |  | 61 |  | 44 |  | 31 |  | n* | 202 | |  |
| Oropharynx | 120 |  | n* |  | n* |  | n* |  | n* |  | n* | 102 | |  |
| **rwEFS Rate** |  |  |  |  |  |  |  |  |  |  |  | **Median rwEFS (95% CI), months** | |  |
| Hypopharynx | 100.00% |  | 29.63% |  | 18.52% |  | 18.52% |  | 9.26% |  | 9.26% | 5.2 (3.5, 10.9) | |  |
| Larynx | 100.00% |  | 43.19% |  | 25.89% |  | 17.87% |  | 15.75% |  | 12.55% | 8.1 (6.6, 11.6) | |  |
| Oral cavity | 100.00% |  | 36.26% |  | 27.96% |  | 25.37% |  | 22.70% |  | 18.90% | 6.9 (5.8, 8.7) | |  |
| Oropharynx | 100.00% |  | 42.46% |  | 34.66% |  | 29.43% |  | 23.67% |  | 19.28% | 8.3 (6.3, 13.5) | |  |
|  | | | | | | | | | | | | | | |

**Abbreviations**: CI, confidence interval; LA HNSCC, locoregionally advanced head and neck squamous cell carcinoma; rwEFS, real-world event-free survival.

* Numbers <11 or numbers that can be used to derive N<11 are not reported to comply with the data suppression policy from SEER-Medicare.

**Supplementary Figure 2. Kaplan-Meier analysis of rwEFS by cancer site among patients with unresected LA HNSCC**

|  | **Months since primary treatment initiation** | | | | | | | | | | | |  |
| --- | --- | --- | --- | --- | --- | --- | --- | --- | --- | --- | --- | --- | --- |
|  |  |  |  |  |  |  |  |  |  |  |  |  | |
| **Months** | **0** |  | **12** |  | **24** |  | **36** |  | **48** |  | **60** | **Total Number of Events** | |
| **Patients at Risk** |  |  |  |  |  |  |  |  |  |  |  |  | |
| Hypopharynx | 107 |  | 31 |  | n* |  | n* |  | n* |  | n* | 91 | |
| Larynx | 440 |  | 226 |  | 127 |  | 84 |  | 57 |  | 39 | 348 | |
| Oral cavity | 404 |  | 145 |  | 79 |  | 50 |  | 35 |  | 19 | 333 | |
| Oropharynx | 603 |  | 278 |  | 181 |  | 140 |  | 96 |  | 66 | 474 | |
| **rwEFS Rate** |  |  |  |  |  |  |  |  |  |  |  | **Median rwEFS (95% CI), months** | |
| Hypopharynx | 100.00% |  | 30.35% |  | 21.54% |  | 16.12% |  | 13.82% |  | 12.67% | 7.0 (5.5, 9.2) | |
| Larynx | 100.00% |  | 52.75% |  | 34.52% |  | 26.14% |  | 22.02% |  | 18.11% | 13.2 (10.8, 16.1) | |
| Oral cavity | 100.00% |  | 38.32% |  | 24.62% |  | 18.01% |  | 15.46% |  | 11.05% | 8.6 (7.7, 9.7) | |
| Oropharynx | 100.00% |  | 47.92% |  | 34.41% |  | 28.74% |  | 24.07% |  | 20.60% | 10.7 (9.4, 13.1) | |
|  | | | | | | | | | | | | | |

**Abbreviations**: CI, confidence interval; LA HNSCC, locoregionally advanced head and neck squamous cell carcinoma; rwEFS, real-world event-free survival.

* Numbers <11 or numbers that can be used to derive N<11 are not reported to comply with the data suppression policy from SEER-Medicare.

**Supplementary Figure 3. Kaplan-Meier analysis of rwEFS by general treatment pattern among patients with resected or unresected LA HNSCC (sensitivity analysis)**

|  |  | **Months since primary treatment initiation** | | | | | | | | | |  |
| --- | --- | --- | --- | --- | --- | --- | --- | --- | --- | --- | --- | --- |
|  |  |  |  |  |  |  |  |  |  |  |  |  |
| **Months** | **0** |  | **12** |  | **24** |  | **36** |  | **48** |  | **60** | **Total Number of Events** |
| **Patients at Risk** |  |  |  |  |  |  |  |  |  |  |  |  |
| Resected | 626 |  | 258 |  | 163 |  | 117 |  | 81 |  | 53 | 507 |
| Unresected | 1554 |  | 780 |  | 449 |  | 312 |  | 218 |  | 144 | 1224 |
| **rwEFS rate** |  |  |  |  |  |  |  |  |  |  |  |  |
| Resected | 100.0% |  | 43.1% |  | 30.3% |  | 24.5% |  | 20.5% |  | 17.1% |  |
| Unresected | 100.0% |  | 52.2% |  | 34.1% |  | 26.4% |  | 22.1% |  | 18.1% |  |

**Abbreviations**: LA HNSCC, locoregionally advanced head and neck squamous cell carcinoma; rwEFS, real-world event-free survival.

**Supplementary Figure 4. Kaplan-Meier analysis of rwOS by cancer site among patients with resected LA HNSCC**

|  | **Months since primary treatment initiation** | | | | | | | | | | |  |
| --- | --- | --- | --- | --- | --- | --- | --- | --- | --- | --- | --- | --- |
|  |  |  |  |  |  |  |  |  |  |  |  |  |
| **Months** | **0** |  | **12** |  | **24** |  | **36** |  | **48** |  | **60** | **Total Number of Events** |
| **Patients at Risk** |  |  |  |  |  |  |  |  |  |  |  |  |
| Hypopharynx | 30 |  | 15 |  | n* |  | n* |  | n* |  | n* | n* |
| Larynx | 221 |  | 150 |  | 94 |  | 66 |  | 45 |  | 25 | 140 |
| Oral cavity | 255 |  | 171 |  | 114 |  | 79 |  | 59 |  | 45 | 149 |
| Oropharynx | 120 |  | 82 |  | 74 |  | 66 |  | 53 |  | 44 | 70 |
| **rwOS Rate** |  |  |  |  |  |  |  |  |  |  |  | **Median rwOS (95% CI), months** |
| Hypopharynx | 100.00% |  | 55.38% |  | 32.22% |  | 27.62% |  | 18.41% |  | 18.41% | 15.6 (4.6, 21.1) |
| Larynx | 100.00% |  | 71.70% |  | 51.95% |  | 43.04% |  | 37.73% |  | 29.52% | 26.2 (19.3, 34.5) |
| Oral cavity | 100.00% |  | 68.70% |  | 53.48% |  | 46.70% |  | 43.46% |  | 39.48% | 30.9 (20.2, 44.8) |
| Oropharynx | 100.00% |  | 70.39% |  | 66.03% |  | 60.60% |  | 52.97% |  | 51.97% | 71.3 (36.9, 87.1) |
|  | | | | | | | | | | | | |

**Abbreviations**: CI, confidence interval; LA HNSCC, locoregionally advanced head and neck squamous cell carcinoma; rwOS, real-world overall survival.

* Numbers <11 or numbers that can be used to derive N<11 are not reported to comply with the data suppression policy from SEER-Medicare.

**Supplementary Figure 5. Kaplan-Meier analysis of rwOS by cancer site among patients with unresected LA HNSCC**

|  | **Months since primary treatment initiation** | | | | | | | | | | |  |
| --- | --- | --- | --- | --- | --- | --- | --- | --- | --- | --- | --- | --- |
|  |  |  |  |  |  |  |  |  |  |  |  |  |
| **Months** | **0** |  | **12** |  | **24** |  | **36** | **42** | **48** | **54** | **60** | **Total Number of Events** |
| **Patients at Risk** |  |  |  |  |  |  |  |  |  |  |  |  |
| Hypopharynx | 107 |  | 59 |  | 42 |  | n* |  | n* |  | n* | 75 |
| Larynx | 440 |  | 332 |  | 235 |  | 168 |  | 108 |  | 82 | 277 |
| Oral cavity | 404 |  | 230 |  | 140 |  | 101 |  | 71 |  | 51 | 277 |
| Oropharynx | 603 |  | 440 |  | 331 |  | 279 |  | 195 |  | 139 | 344 |
| **rwOS Rate** |  |  |  |  |  |  |  |  |  |  |  | **Median rwOS (95% CI), months** |
| Hypopharynx | 100.00% |  | 57.94% |  | 43.99% |  | 38.55% |  | 32.44% |  | 32.44% | 18.6 (11.4, 30.0) |
| Larynx | 100.00% |  | 77.91% |  | 62.89% |  | 50.73% |  | 41.70% |  | 37.03% | 36.9 (31.2, 40.8) |
| Oral cavity | 100.00% |  | 60.10% |  | 42.65% |  | 35.49% |  | 31.11% |  | 28.81% | 17.0 (14.2, 21.3) |
| Oropharynx | 100.00% |  | 75.48% |  | 62.11% |  | 55.57% |  | 49.08% |  | 43.24% | 46.6 (38.2, 55.8) |
|  | | | | | | | | | | | | |

**Abbreviations**: CI, confidence interval; LA HNSCC, locoregionally advanced head and neck squamous cell carcinoma; rwOS, real-world overall survival.

* Numbers <11 or numbers that can be used to derive N<11 are not reported to comply with the data suppression policy from SEER-Medicare.

**Supplementary F****igure 6. Time to recurrence among patients with resected LA HNSCC**

0.00

0.25

0.50

0.75

1.00

0

1

2

3

4

5

6

7

8

9

**Years since primary treatment initiation**

**Cumulative Incidence**

| **Years** | **0** | **1** | **2** | **3** | **4** | **5** | **6** | **7** | **8** | **9** | **Median** |
| --- | --- | --- | --- | --- | --- | --- | --- | --- | --- | --- | --- |
| **Cumulative Incidence** | 0.0% | 41.2% | 48.4% | 51.5% | 53.8% | 56.1% | 57.8% | 58.8% | 59.6% | 60.8% | 2.3 months |
| **Patients at Risk** | 626 | 237 | 151 | 110 | 76 | 50 | 28 | n* | n* | n* |  |

**Abbreviation**: LA HNSCC, locoregionally advanced head and neck squamous cell carcinoma.

* Numbers <11 or numbers that can be used to derive N<11 are not reported to comply with the data suppression policy from SEER-Medicare.

**Supplementary Figure 7. Time to recurrence among patients with unresected LA HNSCC**

0.00

0.25

0.50

0.75

1.00

0

1

2

3

4

5

6

7

8

9

**Years since primary treatment initiation**

**Cumulative Incidence**

| **Years** | **0** | **1** | **2** | **3** | **4** | **5** | **6** | **7** | **8** | **9** | **Median** |
| --- | --- | --- | --- | --- | --- | --- | --- | --- | --- | --- | --- |
| **Cumulative Incidence** | 0.0% | 34.6% | 44.4% | 49.1% | 51.6% | 54.1% | 55.7% | 56.8% | 57.5% | 58.1% | 3.3 months |
| **Patients at Risk** | 1554 | 680 | 408 | 288 | 200 | 133 | 85 | 58 | 37 | n* |  |

**Abbreviation**: LA HNSCC, locoregionally advanced head and neck squamous cell carcinoma.

* Numbers <11 or numbers that can be used to derive N<11 are not reported to comply with the data suppression policy from SEER-Medicare.

**Supplementary Figure 8. Time to recurrence by cancer site among patients with resected LA HNSCC**

0.00

0.25

0.50

0.75

1.00

0

1

2

3

4

5

6

7

8

9

**Years since primary treatment initiation**

**Cumulative Incidence**

Hypopharynx

Larynx

Oral cavity

Oropharynx

| **Years** | **0** | **1** | **2** | **3** | **4** | **5** | **6** | **7** | **8** | **9** | **Median** |
| --- | --- | --- | --- | --- | --- | --- | --- | --- | --- | --- | --- |
| **Cumulative Incidence** |  |  |  |  |  |  |  |  |  |  |  |
| Hypopharynx | 0.0% | 37.0% | 40.7% | 40.7% | 45.4% | 45.4% | 45.4% | 45.4% | 45.4% | 45.4% | - |
| Larynx | 0.0% | 36.0% | 46.2% | 51.4% | 52.8% | 53.7% | 54.9% | 54.9% | 54.9% | 0.0% | 2.4 |
| Oral cavity | 0.0% | 47.1% | 52.3% | 52.9% | 54.2% | 56.5% | 58.6% | 60.2% | 60.2% | 63.3% | 1.6 |
| Oropharynx | 0.0% | 39.0% | 45.9% | 51.1% | 55.9% | 60.3% | 62.9% | 64.8% | 67.3% | 67.3% | 2.7 |
| **Patients at Risk** |  |  |  |  |  |  |  |  |  |  |  |
| Hypopharynx | 30 | n* | n* | n* | n* | n* | n* | n* | n* | n* |  |
| Larynx | 221 | 91 | 46 | 29 | n* | n* | n* | n* | n* | n* |  |
| Oral cavity | 255 | 89 | 61 | 44 | 31 | n* | n* | n* | n* | n* |  |
| Oropharynx | 120 | 49 | n* | n* | n* | n* | n* | n* | n* | n* |  |

**Abbreviation**: LA HNSCC, locoregionally advanced head and neck squamous cell carcinoma.

* Numbers <11 or numbers that can be used to derive N<11 are not reported to comply with the data suppression policy from SEER-Medicare.

**Supplementary Figure 9. Time to recurrence by cancer site among patients with unresected LA HNSCC**

0.00

0.25

0.50

0.75

1.00

0

1

2

3

4

5

6

7

8

9

**Years since primary treatment initiation**

**Cumulative Incidence**

Hypopharynx

Larynx

Oral cavity

Oropharynx

| **Years** | **0** | **1** | **2** | **3** | **4** | **5** | **6** | **7** | **8** | **9** | **Median** |
| --- | --- | --- | --- | --- | --- | --- | --- | --- | --- | --- | --- |
| **Cumulative incidence** |  |  |  |  |  |  |  |  |  |  |  |
| Hypopharynx | 0.0% | 41.9% | 47.8% | 51.1% | 52.3% | 53.4% | 55.0% | 55.0% | 55.0% | 55.0% | 2.6 |
| Larynx | 0.0% | 31.4% | 44.1% | 49.1% | 51.8% | 54.9% | 56.8% | 57.4% | 58.1% | 59.0% | 3.3 |
| Oral cavity | 0.0% | 34.9% | 43.0% | 48.2% | 49.9% | 53.9% | 55.2% | 55.2% | 55.2% | 55.2% | 4.3 |
| Oropharynx | 0.0% | 35.5% | 45.0% | 49.5% | 52.6% | 54.0% | 55.8% | 58.1% | 59.1% | 59.8% | 3.1 |
| **Patients at Risk** |  |  |  |  |  |  |  |  |  |  |  |
| Hypopharynx | 107 | 31 | n* | n* | n* | n* | n* | n* | n* | n* |  |
| Larynx | 440 | 226 | 127 | 84 | 57 | 39 | 26 | n* | n* | n* |  |
| Oral cavity | 404 | 145 | 79 | 50 | 35 | 19 | n* | n* | n* | n* |  |
| Oropharynx | 603 | 278 | 181 | 140 | 96 | 66 | 40 | 29 | n* | n* |  |

**Abbreviation**: LA HNSCC, locoregionally advanced head and neck squamous cell carcinoma

* Numbers <11 or numbers that can be used to derive N<11 are not reported to comply with the data suppression policy from SEER-Medicare.
